# Functional Comparison of *Clostridium butyricum* and Sodium Butyrate Supplementation on Growth, Intestinal Health, and the Anti-inflammatory Response of Broilers

**DOI:** 10.3389/fmicb.2022.914212

**Published:** 2022-06-02

**Authors:** Ling Liu, Huayun Ling, Wei Zhang, Ying Zhou, Youguo Li, Nan Peng, Shumiao Zhao

**Affiliations:** ^1^State Key Laboratory of Agricultural Microbiology, Hubei Hongshan Laboratory, College of Life Science and Technology, Huazhong Agricultural University, Wuhan, China; ^2^Wuhan Sunhy Biology Co., Ltd., Wuhan, China

**Keywords:** *Clostridium butyricum*, sodium butyrate, broiler, growth performance, intestinal health, anti-inflammatory response, antibiotic

## Abstract

Butyrate has been reported to promote proliferation of colonic epithelial cells and maintain intestinal barrier integrity in broilers. Although supplementation of *Clostridium butyricum* and sodium butyrate have been shown to confer benefits on broilers, their effects and mechanisms have not been compared. In this study, *C. butyricum* and sodium butyrate were added into the basal diet of broilers and their effects on growth performance, intestinal health, and anti-inflammatory response were analyzed. It was found that both *C. butyricum* and sodium butyrate showed good probiotic effects on broilers. Their effects on growth rate and expression of inflammation related genes were superior to that of the antibiotic oxytetracycline. Besides, the two dietary supplements improved intestinal structure integrity and secretion of inflammatory cytokines, whereas the antibiotic had negative effects. Comparison of the two supplements revealed that sodium butyrate more effectively improved the growth and intestinal structure of broilers than *C. butyricum*. On the contrary, *C. butyricum* was superior to sodium butyrate in promoting tight junction protein expression and anti-inflammatory response. In summary, this study demonstrates the positive effects of *C. butyricum* and sodium butyrate on broilers, and will serve as a reference for selection of appropriate butyrate supplementation for broilers in the breeding industry.

## Introduction

Short-chain fatty acids (SCFAs), a class of organic acids that includes acetate, propionate, and butyrate, are important metabolites produced by intestinal microbial anaerobic fermentation (Fan et al., 2015). Numerous studies currently show that SCFAs exert diverse functions on the host (Koh et al., 2016). SCFAs, for example, contribute to the integrity of the gut structure by providing a significant portion of energy for colonic epithelial cells (Brahe et al., 2013). Additionally, SCFAs promote intestine health in a variety of other ways, including phagocytosis, intestinal dynamic balance, and immune regulation (Tan et al., 2014). Butyrate, a typical C_4_ SCFA, has garnered much attention in recent years for its beneficial effects on intestinal health (Guilloteau et al., 2010). It is the most important energy substrate for colon cells, promoting epithelial cell growth and differentiation (Jacobi and Odle, 2012; Chen et al., 2015). Some studies have also shown that butyrate, as an anti-inflammatory agent, plays an essential role in modulating immune response and intestinal barrier function (Hamer et al., 2008; Tan et al., 2014). Based on its positive effects on the host, including butyric acid into dairy diets to promote animal growth has become a widely adopted strategy in the feeding industry.

*Clostridium butyricum* spores and sodium butyrate are the two most common butyric acid supplements in the market. *C. butyricum*, a butyric acid-producing anaerobic bacterium, is widely distributed in animal gut and the natural environment (Cassir et al., 2016). Due to its beneficial properties, *C. butyricum* has been recognized as one typical probiotic on a global scale. It has been shown in previous research to significantly improve broiler growth performance, nutritional metabolism, intestinal morphology, and intestinal immune dynamic balance (Takahashi et al., 2018; Huang et al., 2019; Molnar et al., 2020). Additionally, it enhances intestinal barrier function and inhibits the inflammasome signaling pathways in weaned piglets challenged with enterotoxigenic *Escherichia coli* K88 (Li et al., 2018). Sodium butyrate, the sodium salt of butyric acid, has been demonstrated to play positive roles in promoting growth performance and intestinal integrity in piglets and broiler chickens (Hu and Guo, 2007; Smulikowska et al., 2009; Piva et al., 2016). Additionally, it has been shown to repair the imbalanced gut flora caused by a high-fat diet in mice (Zhou et al., 2017).

Although the effects of *C. butyricum* and sodium butyrate on animal’s growth performance and intestinal health have been extensively investigated (Zhang et al., 2009; Zhao et al., 2020), the similarities and differences in their beneficial function on broilers remain unknown. In this study, we detected the impact of *C. butyricum* and sodium butyrate on broilers simultaneously. Results indicated that both two supplements exerted probiotic functions. Specifically, sodium butyrate promoted growth better, while *C. butyricum* was more beneficial for tight junction protein expression and anti-inflammatory response. Comparing their probiotic prosperities could help understand the differences between them and guide the market to choose proper butyric acid supplement.

## Materials and Methods

### Ethics Statement

All experiments were performed in accordance with the ethical standards of Huazhong Agriculture University’s Laboratory Animal Center (HZAUCH-2019-008).

### Experimental Design

A total of 360 one-day-old Cobb500 broilers were randomly assigned to four groups with six replicates per group and 15 broilers per replicate. These four groups were set as follows: basal diet (Control) and basal diet supplemented with 100 g/t (1.0 × 10^9^ CFU/g) *C. butyricum* spores (CB), 500 g/t sodium butyrate (SB), or 200 g/t oxytetracycline (Antibiotic), respectively. Table 1 summarizes the composition and nutritional content of the basal diet. Broilers were obtained from Yicheng Xiangda agriculture and animal husbandry Co., Ltd. *C. butyricum* and SB were obtained from Wuhan Sunhy Biology Co., Ltd. Oxytetracycline was obtained from Henan Xinxiang Huaxu Trading Co., Ltd. All the reagents used in the basal diet were obtained from Hubei Zhonghui agriculture and animal husbandry Co., Ltd.

**TABLE 1:** Nutrients levels and composition of basic diets.

Item (% unless noted)	Starter (1–21 d)	Grower (22–42 d)
**Ingredients**		
Corn (7.8%, crude protein)	530	540
Soybean meal (43%, crude protein)	360	342.50
Rapeseed meal	0	20
Fish powder (68%)	30	0
Soybean oil	40	60
Limestone	12	13
CaHPO_4_	14	13
Lysine (70%)	4	2.80
Methionine	2	1.50
Salt	3	3
Choline Chloride (50%)	1	1
Rice bran	0.80	0
Multivitamin*	2	2
Multimineral^†^	1.20	1.20
Total	1,000	1,000
**Calculated nutrient levels**		
Metabolic energy (kcal/kg)	3,030	3,150
Crude protein	22	20
Total phosphorus	0.69	0.60
Available phosphorus	0.45	0.35
Calcium	1	0.88
Lysine	1.45	1.20
Methionine	0.55	0.45
Methionine +Cysteine	0.88	0.78
Threonine	0.92	0.84
Tryptophan	0.28	0.25
Arginine	1.20	1.12

*Supplied per kilogram of diet: retinyl acetate 5,000–10,000 KIU; vitamin D3 2,000–5,000 KIU; DL-α-tocopheryl acetate ≥ 25,000 mg; menadione ≥ 2,400 mg; thiamine nitrate ≥ 2,000 mg; riboflavin ≥ 6,000 mg; vitamin B6 ≥ 3,500 mg; cyanocobalamin ≥ 12 mg; nicotinamide ≥ 30,000 mg; D-biotin ≥ 75 mg; D-calcium pantothenate ≥ 8,000 mg; folic acid ≥ 950 mg. ^†^Supplied per kilogram of diet: copper 6,000–18,000 mg; iron 30,000–150,000 mg; manganese 60,000–125,000 mg; zinc 50,000–100,000 mg; iodine 400–900 mg; selenium 150–300 mg.

Broilers were raised in wire cages with sufficient water supply, and the room was controlled at standard conditions of temperature, humidity and ventilation (Wu et al., 2019). Before the experiment, the equipment was cleaned and disinfected, particularly, it was fumigated with potassium permanganate and formaldehyde after cleaning and drying. Immunization and deworming procedures were conducted concurrently with the farm routine. Continuous feeding three times daily at a set time was conducted. Throughout the trial period, the feeding and health of broilers were monitored and documented. The breeding experiment was conducted at Sunhy Biology Co., Ltd’s Huanghu breeding facility.

### Sample Collection

Before the experiment started, twelve broilers (two from each replicate) were selected at random from each group to be weighed. The feeding of an additional twelve broilers (selected as above) was stopped at 9:00 p.m. on the 21st and 42nd day of the experiment, respectively, and their water supply was stopped at 7:00 a.m. on the 22nd and 43rd day. Their body weight was recorded and then they were killed. The abdominal cavity was rapidly opened to separate jejunum, ileum, and cecum. Samples of jejunum, ileum, and cecum (about 1–2 cm from the midpoint) were fixed with 4% paraformaldehyde for tissue section preparation and examination of intestinal morphology. Jejunum was cut, washed with sterile normal saline, scraped, and then frozen at −80°C for DNA extraction and gene expression analysis. Ileum and cecal chyme were collected and stored at −20°C for subsequent analysis of volatile SCFAs. Meanwhile, cecum digested samples were taken out and stored at −80°C for 16S rDNA high-throughput sequencing.

### Growth Performance Measurement

Throughout the study, the daily feed consumption, body weight, and health of broilers were all documented. The following formulas were used to determine feed intake (FI), body weight gain (BWG), and feed to gain ratio (F/G), respectively.


FI⁢(g/d⁢⋅⁢bird)=∑

[(Feed⁢amount-Residual⁢amount)/Number⁢of⁢broilers]/Days

BWG⁢(kg/d⋅bird)

=(Final⁢average⁢weight-Initial⁢average⁢weight)/Days

F/G=Total⁢feed⁢consumption/Total⁢weight⁢gain

### Analysis of Intestinal Histomorphology

Jejunum, ileum, and cecum segments were fixed with formaldehyde and embedded in paraffin. Consecutive sections (5 mm) were stained with eosin-methylene blue for morphological observation using an optical microscope (Shinde et al., 2019). From each section, fifteen villi were randomly selected and their villi height (V) and crypt depth (C) were measured. V refers to the distance from the apex of the villus to the entrance of the crypt, while C refers to the distance between the base of the villus and the basal mucosa. The following formula was used to determine the villi height to crypt depth ratio (V/C).


V/C=Villi⁢height/Crypt⁢depth

### 16S rDNA Sequencing

The variable region V3 of the cecal microflora 16S rDNA gene was sequenced using 454 high-throughput sequencing technology with primer pair 341F/534R (Table 2). The software (Mothur) was used to remove the low-quality DNA sequences, and then the distance between the sequences was calculated. Operational taxonomic units (OTUs) were determined as filtered sequencing clusters with 97% similarity level. The microbial diversity of various treatments was investigated (Zhang Z. et al., 2020) and compared using the Saliva and RPD databases. Sangon Biotech (Shanghai) Co., Ltd., performed the sequencing.

**TABLE 2:** Primers used in this study.

Primers	Sequence (5’–3’)
ZO-1-F	TCGGGTTGTGGACACGCTAT
ZO-1-R	TTCATAGGCAGGGAACTTTGTCT
Occludin-F	GTTCCTCATCGTCATCCTGCTC
Occludin-R	CGTTCTTCACCCACTCCTCCAC
TAK1-F	ATGATAATGATTGTCCTACTGCCCC
TAK1-R	GGCAGGCTCAAATGGTAGGC
NF-kB-F	ATGCTCACAGCTTGGTGGGTAA
NF-kB-R	TCATGCGTGTTTCCAGAGTTTC
IL-1β-F	ATGACCAAACTGCTGCGGAG
IL-1β-R	AAGGACTGTGAGCGGGTGTAG
1L-6-F	GGTGATAAATCCCGATGAAGTGG
1L-6-R	AGGCACTGAAACTCCTGGTCTT
TNF-α-F	GGAATGAACCCTCCGCAGTA
TNF-α-R	GCAACAACCAGCTATGCACCC
β-actin-F	CTGACTGACCGCGTTACTCC
β-actin-R	TTGCACATACCGGAGCCATT
341F	CCTACGGGAGGCAGCAG
534R	TAGATTACCGCGGCTGCT

### Determination of Short-Chain Fatty Acids Concentrations

Gas chromatography-mass spectrometry (GC-MS) was used to determine the concentrations of standard solutions and volatile SCFAs in ileum and cecum chyme (Zhang C. et al., 2020). Acetic acid, propionic acid, or butyric acid were dissolved in ether to form standard solutions of varying concentrations. A 50 mg of chyme was dissolved in a mixture of 50 μL phosphoric acid (15%), 100 μL isohexanoic acid (125 μg/mL), and 400 μL ether. The sample was then vortexed and centrifuged at 13,000 *g* for 10 min at 4°C. For analysis, the supernatant was injected into the chromatographic column. SCFAs were analyzed using a Thermo TRACE 1310-ISQ GC-MS system, equipped with an Agilent HP-INNOWAX column (30 m × 0.25 mm ID × 0.25 μm). The split injection was carried out using a 1 μL injection volume (split ratio 10:1). The inlet and transmission line temperature were 250°C, the ion source temperature was 230°C, and the quadrupole temperature was 150°C. The carrier phase was helium at a flow rate of 1.0 mL/min. MS was carried out using an electron bombardment ionization (EI) source and SIM scanning mode. The electron energy was 70 eV.

### Expression of Intestinal Inflammatory Factors and Tight Junction Protein Genes

RNA from jejunum samples was extracted, cDNA was then obtained by reverse transcription from total RNA. Occludin, ZO-1, TAK1, NF-kB, IL-1β, IL-6, and TNF-α gene expression levels were then determined by real-time PCR (qPCR). β-actin gene was used as the reference gene. RT-PCR was performed using the PrimeScript™ RT reagent kit, and qPCR was performed using the SYBR^®^ Premix Ex Taq™ II kit (TAKARA, Biotechnology Co., Ltd., Japan). Table 2 lists all of the primers used.

### Data Analysis

SPSS 16.0 (SPSS Inc., Chicago, IL, United States) was used for variance analysis. Duncan was used for multiple comparisons. Data are expressed as means ± SDs. *p* < 0.05 was considered to be statistically significant. The diversity of the bacterial community was compared using one-way analysis of variance (ANOVA) followed by Tukey *post hoc* test. Non-Metric Multidimensional Scaling (NMDS) analyses and non-parametric multivariate analysis of variance (ADONIS) were performed to estimate the extent differences between the groups, and the significance levels by calculating the weighted and unweighted UniFrac distance matrix using QIIME.

## Results

### Sodium Butyrate Promoted Growth Performance More Effectively Than *Clostridium butyricum*

The F/G ratio of birds fed the four diets (Control, CB, SB, Antibiotic) was maintained at 1.42–1.47 over 1–21 d (Table 3). However, when the feed additive (*C. butyricum*, sodium butyrate, or oxytetracycline) was introduced, the value significantly decreased (*p* < 0.05) from 2.05 to 1.90–1.92 during 22–42 d and from 1.86 to 1.74–1.75 during 1–42 d (Table 3). Specifically, the SB group’s BWG significantly increased (*p* < 0.05) from 56.31 to 62.16 g over the 22–42 d and from 42.13 to 45.76 g throughout the overall period (1–42 d), while FI remained constant in the Control group (Table 3). Although *C. butyricum* supplementation did not affect BWG in the CB group, FI was significantly decreased (*p* < 0.05) from 115.68 to 106.57 g during 22–42 d and from 78.38 to 73.73 g during 1–42 d (Table 3). Besides, there were no significant differences (*p* > 0.05) in BWG and FI between the Antibiotic group and the other two groups (CB, SB), respectively (Table 3). When compared to the CB group, the addition of sodium butyrate significantly increased BWG and FI (*p* < 0.05) throughout 22–42 d and the overall period (1–42 d), but there were no discernable changes in F/G (*p* > 0.05, Table 3).

**TABLE 3:** Effects of diet on the growth of broilers.

	Control	CB	SB	Antibiotic
1–21 d				
FI	41.09 ± 0.97	40.89 ± 0.91	41.54 ± 0.79	40.95 ± 1.06
BWG	27.94 ± 0.69^b^	28.82 ± 0.84^ab^	29.37 ± 0.98^a^	28.78 ± 0.61^ab^
F/G	1.47 ± 0.06	1.42 ± 0.06	1.42 ± 0.04	1.42 ± 0.04
22–42 d				
FI	115.68 ± 5.95^a^	106.57 ± 7.91^b^	118.80 ± 6.32^a^	114.85 ± 5.77^a^
BWG	56.31 ± 2.75^b^	56.16 ± 3.30^b^	62.16 ± 5.27^a^	60.45 ± 3.76^ab^
F/G	2.05 ± 0.04^a^	1.90 ± 0.08^b^	1.92 ± 0.07^b^	1.90 ± 0.04^b^
1–42 d				
FI	78.38 ± 3.19^a^	73.73 ± 3.81^b^	80.17 ± 3.52^a^	77.90 ± 3.31^a^
BWG	42.13 ± 1.32^b^	42.49 ± 1.96^b^	45.76 ± 2.71^a^	44.61 ± 2.16^ab^
F/G	1.86 ± 0.04^a^	1.74 ± 0.05^b^	1.75 ± 0.04^b^	1.75 ± 0.04^b^

*FI, feed intake (g/d⋅bird); BWG, body weight gain (kg/d⋅bird); F/G, feed to gain ratio.*

### *Clostridium butyricum* and Sodium Butyrate Improved Intestinal Structural Integrity

**FIGURE 1:**
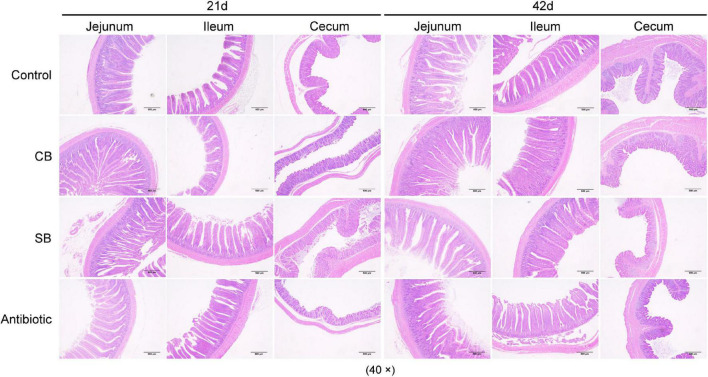
Intestinal tissue morphology. Samples of jejunum, ileum, and cecum (approximately 1–2 cm obtained from the midpoint) at 21 and 42 d were fixed. Villi and goblet cells were observed from eosin-methylene blue-stained sections of samples by optical microscopy at 40×.

As shown in Table 4, oxytetracycline treatment had no significant effects on villi structure at 21 d. At 42 d, however, significantly reduced V/C suggested that the intestinal structure and morphology were damaged (Figure 1 and Table 4). Broilers fed with diet supplemented with CB or SB, had longer, wider villi throughout the overall period (Figure 1 and Table 4). After 21 days, V and V/C of jejunum and cecum in the CB and SB groups were both significantly increased (*p* < 0.05) (Figure 1 and Table 4). Specifically, V of jejunum samples was increased from 711.73 to 987.79 μm and 985.14 μm in CB and SB, respectively, while that of cecum increased from 143.95 to 166.58 μm and 187.70 μm at the same time (Table 4). V/C increased from 4.88 to 7.62 and 8.41 in jejunum, and from 1.13 to 1.45 and 1.74 in cecum samples in CB and SB, respectively (Table 4). Furthermore, V/C of ileum was increased (*p* < 0.05) from 4.07 to 6.70 and 7.08, with significant statistical differences, and C was reduced from 127.23 to 76.58 μm in the CB group and 100.37 μm in the SB group (*p* < 0.05, Table 4). At 42 d, we observed that V/C of jejunum, ileum, and cecum were almost all significantly increased (*p* < 0.05) in the CB and SB diets (Table 4). In particular, V/C of jejunum increased from 5.43 to 6.93 in the SB group, that of ileum increased from 5.39 to 6.58 μm and 6.73 μm, while that of cecum samples increased from 0.93 to 1.50 and 1.75 (Table 4). Furthermore, it was clear that V/C in the SB group increased more apparent than in the CB group, not only in various segments of small intestine but also throughout different development stages of broilers (Table 4).

**TABLE 4:** Effects of diet on structure of intestinal villi in broilers.

		Control	CB	SB	Antibiotic
21 d					
Jejunum	V	711.73 ± 124.66^b^	987.79 ± 124.66^a^	985.14 ± 221.50^a^	628.08 ± 62.30^b^
	C	151.82 ± 46.53^ab^	129.24 ± 19.89^b^	120.60 ± 33.68^b^	173.74 ± 27.38^a^
	V/C	4.88 ± 0.91^b^	7.62 ± 1.06^a^	8.41 ± 1.77^a^	3.68 ± 0.71^b^
Ileum	V	549.93 ± 63.83^b^	504.15 ± 26.07^b^	705.65 ± 132.81^a^	523.35 ± 38.44^b^
	C	127.23 ± 12.19^a^	76.58 ± 9.15^c^	100.37 ± 11.83^b^	132.22 ± 12.22^a^
	V/C	4.07 ± 0.24^b^	6.70 ± 1.15^a^	7.08 ± 1.37^a^	4.00 ± 0.58^b^
Cecum	V	143.95 ± 25.52^b^	166.58 ± 27.15^ab^	187.70 ± 17.85^a^	140.79 ± 11.51^b^
	C	128.07 ± 20.29	115.80 ± 15.16	111.82 ± 28.91	142.61 ± 29.28
	V/C	1.13 ± 0.20^bc^	1.45 ± 0.27^ab^	1.74 ± 0.34^a^	1.03 ± 0.27^c^
42 d					
Jejunum	V	1,015.09 ± 176.38^bc^	1,138.61 ± 157.01^ab^	1,201.12 ± 92.46^a^	903.92 ± 102.50^c^
	C	185.74 ± 17.82^b^	279.79 ± 38.46^a^	184.77 ± 58.36^b^	246.79 ± 54.33^a^
	V/C	5.43 ± 0.59^b^	4.11 ± 0.67^c^	6.93 ± 1.63^a^	3.79 ± 0.82^c^
Ileum	V	754.30 ± 119.87^b^	936.03 ± 129.26^a^	795.69 ± 113.05^b^	672.43 ± 78.85^b^
	C	142.10 ± 27.63^ab^	145.51 ± 31.00^ab^	120.57 ± 29.15^b^	173.00 ± 31.22^a^
	V/C	5.39 ± 0.83^b^	6.58 ± 1.29^a^	6.73 ± 1.18^a^	4.07 ± 1.10^c^
Cecum	V	152.05 ± 36.18^b^	195.83 ± 10.99^a^	193.05 ± 18.61^a^	147.59 ± 19.09^b^
	C	165.19 ± 40.80^ab^	135.46 ± 27.13^bc^	111.43 ± 13.46^c^	174.54 ± 33.77^a^
	V/C	0.93 ± 0.13^b^	1.50 ± 0.35^a^	1.75 ± 0.24^a^	0.88 ± 0.23^b^

*V, villus height (μm); C, crypt depth (μm); V/C, villus height to crypt depth.*

**FIGURE 2:**
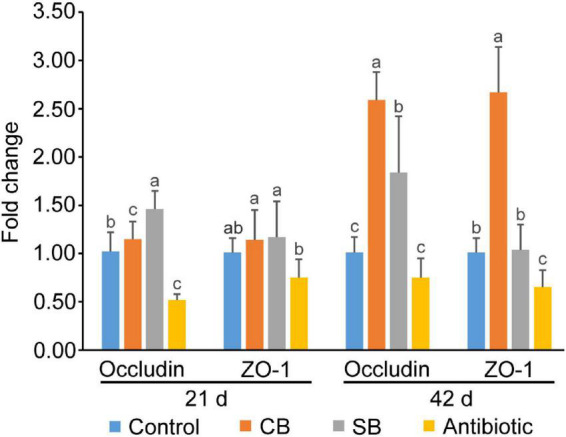
Gene expression of tight junction proteins in broiler jejunum. Significant differences are shown by bars labeled with various letters.

Real-time PCR findings revealed that broilers fed the SB diet had higher levels of Occludin gene expression (*p* < 0.05) than those in the Control group at all stages (Figure 2). Furthermore, *C. butyricum* supplementation significantly increased gene expression levels of both Occludin and ZO-1 (*p* < 0.05) at 42 d (Figure 2). However, all tight junction protein genes were lowly expressed in the oxytetracycline-supplemented group, with Occludin levels falling by 26–49%, and ZO-1 levels falling by 26–36% compared to the Control group (Figure 2).

### *Clostridium butyricum* and Sodium Butyrate Regulated Intestinal Microbial Community Structure

**FIGURE 3:**
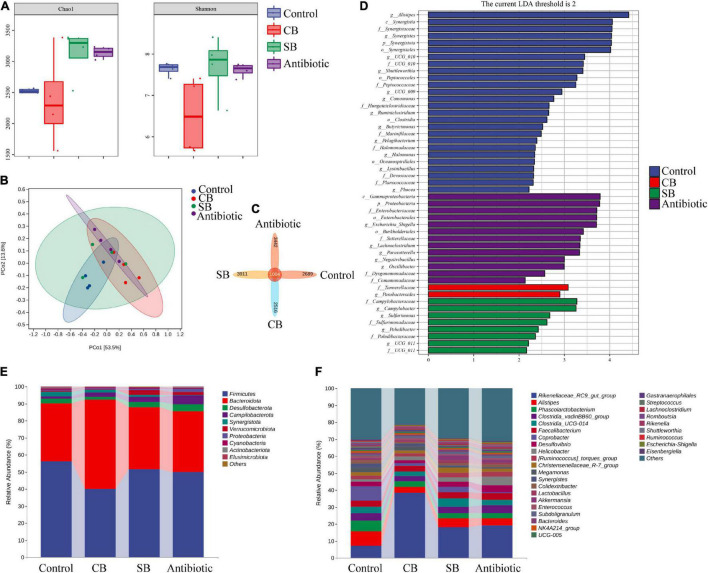
Effects of different feed additives on caeca microbial community. **(A)** Alpha diversity was assessed by Chao1 and Shannon indexes. **(B)** Principal coordinates analysis (PCoA) based on UniFrac distance. **(C)** OTU Venn diagram. **(D)** Linear discriminant analysis (LDA) scores for various taxa abundances. **(E)** Microbial composition at the phylum level is shown as relative abundance. **(F)** Microbial composition at the genus level is shown as relative abundance.

Chao1 and Shannon indexes were used to express the alpha diversity of microbial community in cecum. The principal coordinates analysis (PCoA) based on UniFrac distance was used to assess the community structure differences. Results indicated that sodium butyrate or oxytetracycline supplementation significantly increased the Chao1 index, but did not change the Shannon index (Figure 3A). However, supplementation of *C. butyricum* had no significant effects on the alpha diversity of intestinal microbial community (Figure 3A). Besides, there were no obvious differences in the beta diversity among these four groups (Figure 3B). According to the Venn diagram, there were 1004 universal OTUs shared by all four groups, as well as 2,689, 2,516, 3,911, and 3,442 unique OTUs in the Control, CB, SB, and Antibiotic group, respectively (Figure 3C). Furthermore, linear discriminant analysis (LDA) showed that *Parabacteroides* was considerably more abundant in the CB group at the genus level, while the addition of SB increased the abundance of *Campylobacter*, *sulfurimonas*, and *Paludibacter* at the genus level (Figure 3D).

At the phylum level, the relative abundance of microbial community revealed that Firmicutes and Bacteroidota were the most abundant orders (Figure 3E). Among them, the proportion of Bacteroidota in the CB group was higher than that in the other three groups (Figure 3E). Besides, SB diet increased the proportion of Verrucomicrobiota significantly (Figure 3E). At the genus level, *Rikenellaceae*, *Alistipes*, and *Coprobacter* were the most predominant orders in the Control group (Figure 3F). The addition of *C. butyricum* produced the most obvious change in microbial structure among the three treatments (Figure 3F). It is noteworthy that the abundance of *Akkermansia* significantly increased in the CB group (Figure 3F). Compared to the other three groups, antibiotic diet decreased the proportion of *Coprobacter* but increased that of *Helicobacter* (Figure 3F).

### *Clostridium butyricum* Promoted Short-Chain Fatty Acids Production More Effectively Than Sodium Butyrate

**FIGURE 4:**
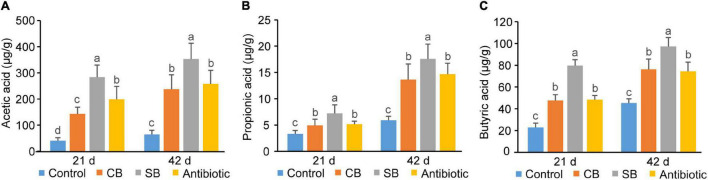
SCFAs concentrations in broiler ileum chyme. **(A)** Acetic acid. **(B)** Propionic acid. **(C)** Butyric acid. SCFAs concentrations are expressed in microgram per gram of chyme sample (μg/g). Significant differences are shown by bars labeled with various letters.

**FIGURE 5:**
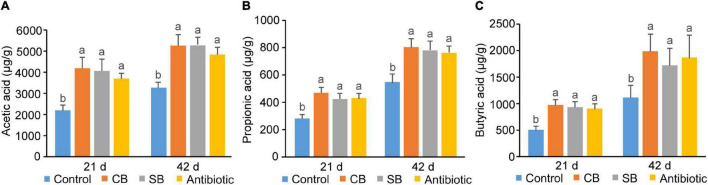
SCFA concentrations in broiler cecum chyme. **(A)** Acetic acid. **(B)** Propionic acid. **(C)** Butyric acid. SCFAs concentrations are expressed as micrograms per gram of chyme sample (μg/g). Significant differences are shown by bars labeled with various letters.

Concentrations of three SCFAs (acetic acid, propionic acid, and butyric acid) in broiler ileum chyme across three supplementation groups (CB, SB, Antibiotic) were significantly higher than those in the Control group (*p* < 0.05, Figure 4). The data showed that sodium butyrate supplementation had the most significant effect on the concentrations of the three SCFAs, while CB diet and antibiotic diet had similar effects on them (Figure 4). In cecum of broilers, the supplementation of *C. butyricum*, sodium butyrate, or oxytetracycline increased concentrations of SCFAs to a similar extent (*p* < 0.05, Figure 5).

### *Clostridium butyricum* Improved Anti-inflammatory Response More Than Sodium Butyrate

**FIGURE 6:**
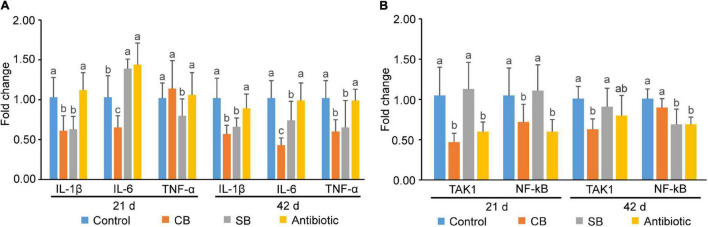
Gene expression of inflammatory and immune-related genes in broiler jejunal mucosa. **(A)** Gene expression of inflammatory cytokines (IL-1β, IL-6, and TNF-α). **(B)** Gene expression of signaling pathway-related proteins (TAK1, NF-kB). Significant differences are shown by bars labeled with various letters.

Results demonstrated that CB diet decreased the expression levels of IL-1β and IL-6 during 1–21 d (*p* < 0.05, Figure 6A) and that of IL-1β, IL-6, and TNF-α during 1–42 d (*p* < 0.05, Figure 6A). Especially, the expression of IL-6 decreased by 2.37 times when compared with the Control group at 42d (Figure 6A). For the SB-supplemented group, other than the expression of IL-6 at 21d which was increased, the rest were all decreased (Figure 6A). Although both *C. butyricum* and sodium butyrate were helpful to broilers, the benefits of *C. butyricum* were more apparent (Figure 6A). In addition, the expression levels of cytokines in the Antibiotic group were comparable to those in the Control group (Figure 6A).

Broilers fed with the CB diet or antibiotic diet exhibited lower gene expression levels of TAK1 and NF-kB (*p* < 0.05) in jejunal mucosa than those fed with the basal diet or SB diet at 21 days of age (Figure 6B). Among them, the addition of *C. butyricum* decreased the gene expression level of TAK1 by 2.23 times (Figure 6B). At 42 d, three treatments (CB, SB, Antibiotic) showed various degrees of inhibitory effects on gene expression of these two genes (Figure 6B). It’s worth mentioning that SB-supplementation decreased the expression of NF-kB at 42 d, while there was no expression inhibition on 21-day old broilers (Figure 6B).

## Discussion

According to the findings (Table 3), there were no significant differences (*p* > 0.05) in broiler growth indicators between the two butyrate-supplemented groups and the oxytetracycline-supplemented group. The probiotic effect of *C. butyricum* and sodium butyrate on growth performance is consistent with earlier findings (Lan et al., 2020), suggesting that these two butyrate supplements could be used as alternatives to growth-promoting antibiotics. Notably, FI in the CB group significantly decreased (*p* < 0.05) throughout the experimental period (1–42 d) (Table 3). We speculated that the vector of supplemented *C. butyricum* affected the taste of feed, and thus reduced the feed intake of broilers. However, butyric acid generated by *C. butyricum* supplied approximately 10–30% of energy needs for broilers (Bergman, 1990), and improved food digestibility by lowering intestinal pH and inhibiting pathogenic bacteria (Hegazy and El-Bedewy, 2010). There have also been reports that *C. butyricum* or sodium butyrate diet has no impact on animal growth performance (Zhang et al., 2011), which may be related to the effective dosage, diet structure, animal health, or even environmental factors.

Compared with the *C. butyricum* group, sodium butyrate significantly increased FI and BWG (*p* < 0.05, Table 3). Sodium butyrate, whose beneficial effects depend largely on dose, can steadily promote growth if taken in adequate quantities. The probiotic effect of *C. butyricum*, on the other hand, will be affected by a variety of factors, including viable count and intestine physiological state. Furthermore, *C. butyricum* in the gut consumes enteral nutrients, reducing growth performance. According to these findings, sodium butyrate is a more stable form of butyrate supplementation for broiler growth performance than *C. butyricum.*

Intestinal health is defined as having a fully functional intestinal structure, intestinal mucosal immune balance, and intestinal microbial balance. In this study, the intestinal morphological structure was damaged by oxytetracycline as shown by decreased V/C at different periods (Figure 1 and Table 4). However, *C. butyricum* and sodium butyrate diets both significantly improved V, C, V/C of jejunum, ileum, and cecum (*p* < 0.05, Table 4), preserving the normal morphology of intestinal epithelial cells (Figure 1). This result is consistent with previous findings (Sikandar et al., 2017). Furthermore, when compared to *C. butyricum*, the findings showed that sodium butyrate was more beneficial for maintaining intact intestinal structure (Table 4). We hypothesized that because sodium butyrate was in close contact with the intestinal tract, it would help to restore the intestinal barrier more rapidly.

Occludin and ZO-1, the major tight junction proteins in the animal gut, are important markers for assessing intestinal permeability and integrity (Zong et al., 2019). Zhao et al. (2020) found that *C. butyricum* and butyrate significantly increased the expression of Occludin, Claudin-1, and ZO-1 in rat model of severe acute pancreatitis with intra-abdominal hypertension. In this study, *C. butyricum* and sodium butyrate both promoted the gene expression of Occludin and ZO-1 in jejunum (Figure 2), resulting in increased formation of tight junction structures and strengthened intestinal barrier, which prevented the passage of toxins and pathogens and decreased disease occurrence. Simultaneously, *C. butyricum* exhibited a more pronounced expression-promoting effect than sodium butyrate (Figure 2). We speculated that *C. butyricum* improved intestinal barrier function more continuously through both live bacteria and its metabolites. However, oxytetracycline supplementation significantly reduced the gene expression levels of tight junction proteins (Figure 2), which is inconsistent with previous report that antibiotic increased tight junction proteins expression in broilers infected with *E. coli* K88 (Zhang et al., 2016). Therefore, the use of antibiotics may be more conducive to the recovery of intestinal barrier infected by pathogenic bacteria, while supplements such as *C. butyricum* and sodium butyrate are more suitable for maintaining normal intestinal health.

There are many and complex microbiotas in the poultry intestine, the composition and diversity of which are affected by a variety of factors (Stanley et al., 2013). Wu et al. (2018) found that 800 mg/kg sodium butyrate significantly decreased the relative abundance of Enterobacteriaceae but increased that of Lachnospiraceae and Rikenellaceae in broilers cecum. In this study, the three supplemented diets did not significantly alter the species diversity and composition of the cecal community, as reflected by the Shannon index, Principal coordinates analysis and Venn diagram (Figures 3A–C). However, the species relative abundance of these four groups differed at different taxonomic levels (Figures 3D–F). Paludibacter, whose abundance increased in the SB group (Figure 3D), has been identified as propionate-producing bacteria (Qiu et al., 2014) and may help to promote gut health via the probiotic function of propionate. *Akkermansia*, a symbiotic-bacteria in the human gut, has been widely studied in recent years for preventing and treating diabetes, obesity and cancer (Cani and de Vos, 2017). Consequently, the increase of Verrucomicrobiota at the phylum level (Figure 3E) and *Akkermansia* at the genus level (Figure 3F) could also explain the probiotic effects of SB diet on broilers. *C. butyricum*, however, was not included in the list of dominating species in the CB group (Figure 3D). We speculated that this species might be viable in broiler gut but does not colonize. *Rikenellaceae*, the main component at the genus level in this study, was similarly increased in the supplemented groups (CB, SB) compared to the Control group (Figure 3F). Because of its high carbohydrate fermentation capacity, this species could generate butyrate and therefore exert a probiotic function on the host (Vander Wyst et al., 2021). When butyrate supplementation was administered, other dominating bacteria, such as *Clostridial* and *Lactobacillus*, which could both generate SCFAs increased (Figure 3F). Furthermore, the detection of SCFAs concentrations revealed that SCFAs in the CB and SB groups were much higher than that in the Control group (Figures 4, 5), supporting the conclusion that SCFAs-producing bacteria were the dominant intestinal microbiota and significantly contributed to host health. *Helicobacter*, closely related to the occurrence of chronic gastritis, gastric ulcer and gastric cancer (Alipour, 2021), increased significantly in the Antibiotic group (Figure 3F). That might be the reason why the supplementation of antibiotic destroyed the normal morphology and structural integrity of the intestine in this study (Figure 1 and Table 4).

TNF-α, IL-1β, and IL-6 are the three main inflammatory cytokines that reflect the host’s inflammatory state (Alvarez et al., 2020). Chen et al. (2018) found that 0.4% *C. butyricum* significantly decreased the expression of TNF-α and increased the expression of the anti-inflammatory cytokine IL-10 in weaned piglet ileum mucosa. Ni et al. (2010) reported that butyric acid up-regulated the production of IL-10 and inhibited the production of TNF-α, IL-1β, and NO. In this study, although there was no significant inflammation in the control group, *C. butyricum* and sodium butyrate both decreased inflammatory cytokines expression (Figure 6A), thus maintaining intestinal tract in a state of low inflammation level. Furthermore, *C. butyricum* decreased the expression of inflammatory cytokines more than sodium butyrate in most cases (Figure 6A), suggesting that *C. butyricum* has more apparent anti-inflammatory benefits than sodium butyrate (Figure 6B). We hypothesized that the numerous SCFAs generated by *C. butyricum*, including not only butyric acid but also acetic acid and propionic acid would have additive effects on the inhabitation of inflammation related genes expression, enhancing the host’s inflammatory regulation. TAK1 is an IKK kinase involved in the NF-kB signaling pathway (Adhikari et al., 2007). NF-kB is an important transcription factor that promotes the expression of a variety of inflammatory and immune-related genes (Zhang et al., 2017). Based on the expression level of inflammatory cytokines, we further detected the expression of genes related to inflammatory signaling pathway. TAK1 and NF-kB expression levels were significantly lower in the treatment groups (CB, SB, Antibiotic) than that in the Control group (*p* < 0.05) (Figure 6B), suggesting that *C. butyricum*, sodium butyrate, and oxytetracycline may all inhibit the activation of the NF-kB pathway to reduce inflammation. Besides, CB supplementation outperformed the SB diet in terms of inhibiting the inflammatory response (Figure 6B).

## Data Availability Statement

The original contributions presented in the study are included in the article/supplementary material, further inquiries can be directed to the corresponding author.

## Ethics Statement

The animal study was reviewed and approved by the Huazhong Agricultural University’s Laboratory Animal Center (HZAUCH-2019-008).

## Author Contributions

LL and HL carried out the animal experiments and data analysis and drafted the manuscript. WZ and YZ participated in the animal trial. YL and NP helped with study design. SZ designed the study and revised the manuscript. All authors contributed to the article and approved the submitted version.

## Conflict of Interest

HL, WZ, and YZ were employed by Wuhan Sunhy Biology Co., Ltd. The remaining authors declare that the research was conducted in the absence of any commercial or financial relationships that could be construed as a potential conflict of interest.

## Publisher’s Note

All claims expressed in this article are solely those of the authors and do not necessarily represent those of their affiliated organizations, or those of the publisher, the editors and the reviewers. Any product that may be evaluated in this article, or claim that may be made by its manufacturer, is not guaranteed or endorsed by the publisher.

## Abbreviations

CB: *Clostridium butyricum*
SB: sodium butyrate
SCFAs: short-chain fatty acids
FI: feed intake
BWG: body weight gain
V: villi height
C: crypt depth
OTUs: operational taxonomic units
GC-MS: gas chromatography-mass spectrometry
EI: electron bombardment ionization
ZO-1: Zonula occludens-1
TAK1: TGF-β activated kinase-1
NF-kB: nuclear factor kappa B
IL-1β: Interleukin-1β
IL-6: Interleukin-6
TNF-α: Tumor necrosis factor-α
PCoA: principal coordinates analysis
LDA: linear discriminant analysis
IKK: inhibitor of nuclear factor kappa B kinase
IL-10: Interleukin-10.
